# Coordinated Priming of NKG2D Pathway by IL-15 Enhanced Functional Properties of Cytotoxic CD4^+^CD28^−^ T Cells Expanded in Systemic Lupus Erythematosus

**DOI:** 10.1007/s10753-023-01860-z

**Published:** 2023-07-06

**Authors:** Tingting Wang, Laiyou Wei, Shuhui Meng, Wencong Song, Yulan Chen, Heng Li, Qianqian Zhao, Zhenyou Jiang, Dongzhou Liu, Huan Ren, Xiaoping Hong

**Affiliations:** 1 Department of Rheumatology and Immunology, The Second Clinical Medical College, Jinan University (Shenzhen People’s Hospital), Shenzhen, 518020 China; 2 Integrated Chinese and Western Medicine Postdoctoral Research Station, Jinan University, Guangzhou, 510632 China; 3 School of Medicine, Southern University of Science and Technology, Shenzhen, 518055 China; 4 Shenzhen People’s Hospital, The Frist Affiliated Hospital of Southern University of Science and Technology, Shenzhen, 518020 China; 5 Department of Microbiology and Immunology, College of Basic Medicine and Public Hygiene, Jinan University, Guangzhou, 510632 China

**Keywords:** interleukin-15-NKG2D, cytotoxic CD4^+^CD28^−^ T cells, inflammation, cytolytic function, tissue damage

## Abstract

Systemic lupus erythematosus (SLE) is a systemic autoimmune disorder, and numerous aberrations of T cell responses have been reported and were implicated in its pathophysiology. Recently, CD4-positive T cells with cytotoxic potential were shown to be involved in autoimmune disease progression and tissue damage. However, the effector functions of this cell type and their potential molecular mechanisms in SLE patients remain to be elucidated. In this study, we find that cytotoxic CD4^+^CD28^−^ T cells are expanded in SLE patients with flow cytometry analysis, and the percentage of CD4^+^CD28^−^ T cells positively correlates with the Systemic Lupus International Collaborating Clinics/ACR Damage Index (SDI). Furthermore, our study suggests that interleukin-15 (IL-15) promotes the expansion, proliferation, and cytotoxic function of CD4^+^CD28^−^ T cells in SLE patients through activation of the Janus kinase3-STAT5 pathway. Further study indicates that IL-15 not only mediates the upregulation of NKG2D, but also cooperates with the NKG2D pathway to regulate the activation of the phosphatidylinositol 3-kinase (PI3K)/protein kinase B (AKT) pathway. Together, our study demonstrated that proinflammatory and cytolytic CD4^+^CD28^−^ T cells expand in SLE patients. The pathogenic potential of these CD4^+^CD28^−^ T cells is driven by the coupling of the IL-15/IL-15R signaling pathway and the NKG2D/DAP10 signaling pathway, which may open new avenues for therapeutic intervention to prevent SLE progression.

**Supplementary Information:**

The online version contains supplementary material available at 10.1007/s10753-023-01860-z.

## INTRODUCTION

Systemic lupus erythematosus (SLE) is a complex autoimmune disorder characterized by an aberrant cytokine milieu as well as by overproduction of pathogenic autoantibodies [1, 2]. SLE constitutes a prototypic systemic disease that affects nearly all organs and tissues, and the kidney is one of the common targets of damage [3, 4]. Various immunological abnormalities related to the disease have been identified, and overriding abnormalities are hyperactivity of B and T lymphocytes [5, 6]. B cells, as antibody-producing cells, have been studied extensively. Moreover, there is an increasing recognition of the critical roles of T cells in the pathogenesis of both SLE and lupus nephritis [7–10]. T cells represent the majority of renal-infiltrating immune cells [11, 12] and are critically involved in the development of inflammation and organ damage. CD4^+^ T cells, including T helper type 1 (Th1), T helper type 2 (Th2), T helper type 17 (Th17), regulatory T cells (Tregs), follicular helper T (Tfh) cells, as helpers and regulators of immune responses, have been reported to initiate and perpetuate inflammation and play a critical role in the immune pathogenesis of SLE and Lupus nephritis [13–17].

CD4^+^ T cells with cytotoxic activity are a specialized CD4^+^ T subset. The existence of these cells has been reported in several autoimmune and chronic inflammatory diseases, including rheumatoid arthritis (RA) [18, 19], multiple sclerosis (MS) [20], acute coronary syndrome (ACS) [21, 22], and IgG4-related disease (IgG4-RD) [23, 24]. CD4^+^ cytotoxic T cells are characterized by a lack of expression of the surface co-stimulatory molecule CD28. Furthermore, these cells were previously shown to express cytotoxic-related genes and share many phenotypic features with CD8^+^ T cells and natural killer (NK) cells [25, 26]. Upon activation, CD4^+^ cytotoxic T cells release perforin, granzymes, and inflammation cytokines. Secretion of this arsenal of chemical weaponry promotes the death of target cells in a number of ways [27]. Together, these functions of CD4^+^ cytotoxic T cells support the idea that the expansion of these cells plays a pathogenic role in disease development. Thus, understanding the complex functions of CD4^+^ cytotoxic T cells should facilitate therapeutic intervention to prevent disease development. However, the highly variable combination of surface biomarkers used to define CD4^+^ cytotoxic T cells, including cytotoxic and regulatory T cell molecule (CRTAM) [28], CX3C motif chemokine receptor 1 (CX3CR1) [20], natural killer group 2D (NKG2D) [29, 30], and SLAM family member 7 (SLAMF7) [24, 31], renders functional comparisons challenging. In SLE, previous studies have shown that CD4^+^CD28^−^ T cells, with the capacity of proinflammatory secretion and higher proliferative activity, were expanded in the peripheral blood of patients with moderately active SLE. The percentage of CD4^+^CD28^−^ T cells positively correlated with the Systemic Lupus International Collaborating Clinics/ACR Damage Index (SDI) [32, 33]. CD4^+^CD28^−^ T cells have previously been reported to predict the occurrence of new lung damage in SLE patients [34]. However, the functional properties and the possible pathogenic involvement of the expanded CD4^+^CD28^−^ T cells in SLE remain to be elucidated.

Here, we set out to characterize the phenotype and function of cytotoxic CD4^+^CD28^−^ T cells in SLE patients using flow cytometry. We investigated the effects of IL-15 on CD4^+^CD28^−^ T cells in SLE patients and inquired into the underlying molecular mechanisms.

## MATERIALS AND METHODS

### Study Subjects

A total of 149 patients with SLE were enrolled from the Department of Rheumatology and Immunology, Shenzhen People’s Hospital, including 80 patients with nephritis (LN group) and 74 patients without nephritis (NLN group). In total, 39 healthy individuals (age and gender-matched) were recruited as controls (Supplementary Table 1). Patients with other autoimmune diseases, infectious diseases, or over 70 years old were excluded from this study. Blood samples were obtained from all patients and healthy controls. All patients fulfilled the Systemic Lupus International Collaborating Clinics (SLICC) classification criteria for SLE [35]. The Systemic Lupus Erythematosus Disease Activity Index 2000 (SLEDAI-2 K) score for patients is calculated using the SLEDAI-2 K score calculator according to both clinical and laboratory parameters (https://qxmd.com/calculate/calculator_335/sledai-2k) [36]. The SDI [37] was also assessed. The diagnosis of LN was confirmed by renal biopsy as per the International Society of Nephrology and Renal Pathology Society (ISN/RPS) criteria [38]. Demographic, clinical, and laboratory data were collected from hospital records or by questionnaire and reviewed by experienced physicians.

### Flow Cytometry

Blood samples were collected in EDTA anticoagulant tube, and 100 µl blood samples were transferred to a new tube for flow cytometry analysis. Peripheral blood mononuclear cells (PBMCs) were isolated using density gradient centrifugation. The frequency of CD4^+^CD28^−^ T cells and their phenotypical characterization were determined by staining blood with antibodies, followed by erythrocyte lysis with Lyse/Fix buffer (BD Biosciences). The percentage of circulating CD4^+^CD28^−^ T cells was calculated as the percentage of CD4^+^ T cells. For intracellular staining, including staining for cytotoxic molecules (granzyme B (GZMB), granzyme A (GZMA), perforin (PRF1)), transcription factor (T-bet, Runx3) and cytokines (interferon-gamma (IFN-γ), tumor necrosis factor-alpha (TNF-α)), PBMCs or cells from whole blood after surface marker staining and erythrocyte lysis, was fixed, permeabilized and stained with antibodies using a Fixation/Permeabilization Solution Kit (BD Biosciences) according to the manufacturer’s instructions. For assessing the phosphorylation state of the signal transducer and activator of transcription 5 (STAT5), CD4^+^CD28^−^ T cells were isolated and then cultured alone or treated with IL-15. Phosphorylated STAT5, Akt, and extracellular signal-regulated kinase (ERK) were assessed via the BD Phosflow method using STAT5 antibody, Akt antibody, ERK1/2 antibody, BD Phosflow Lyse/Fix Buffer, and BD Perm Buffer III (all BD Biosciences) as per the manufacturer’s instructions.

Antibodies targeting the following molecules were used in this study: CD3 (clone: HIT3a; PerCP/Cy5.5 BioLegend, clone: UCHT1; Brilliant Violet421 BioLegend), CD4 (clone: A161A1; APC-Cy7, PE/Cyanine7 BioLegend), CD8 (clone: SK1; APC-Cy7 Biolegend and BD Biosciences), CD28 (clone: CD28.2; FITC, PE-Cy7 BioLegend), granzyme B (clone: QA18A28; PE Biolegend, clone: QA16A02; Alexa Fluor 647 Biolegend), granzyme A (clone: CB9; PE Biolegend), perforin (clone: dG9; PE/Cyanine7 BioLegend), CX3CR1 (clone: 2A9-1; APC eBioscience), NKG2D (clone: 1D11; APC BD Biosciences), T-bet (clone: 4B10; PE/Cyanine7 BioLegend), Runx3 (clone: R3-5G4; PE BD Biosciences), TNF-α (clone: MAb11; APC BioLegend), IFN-γ (clone: 4S.B3; PE BioLegend and BD Biosciences, PE/Cyanine7 BD Biosciences), STAT5 (pY694; PE BD Biosciences), AKT (pS473; Alexa Fluor 647 BD Biosciences), ERK1/2 (pT202/pY204; PE/Cyanine7 BD Biosciences). Data were acquired using FCAS Canto II (BD Biosciences) and analyzed with FlowJo software.

### Intracellular Cytokine Staining

An intracellular cytokine assay was performed to assess cytokine levels. Briefly, PBMCs were stimulated with a combination of phorbol 12-myristate 12-acetate (PMA) and ionomycin (both from Sigma-Aldrich) for 4 h. Three hours prior to analysis, Brefeldin A (Sigma-Aldrich) was added. PBMCs were stimulated in a standard culture medium without PMA and ionomycin (Ion) as a control. To analyze IFN-γ and TNF-α producing cells, the cell surface of PBMCs was stained with antibodies (anti-human CD3, anti-human CD4, anti-human CD28). Next, cells were fixed, permeabilized, and stained with antibodies (anti-human IFN-γ and anti-human TNF-α).

### Degranulation Assay

PBMCs were isolated from 10 ml fresh peripheral blood samples by Ficoll-Paque (GE Healthcare, USA) density gradient centrifugation. To detect surface mobilization of lysosomal associated membrane protein-1 (CD107a), a marker of degranulation associated with cytolytic function, PBMCs were seeded in 96-well plates and cultured alone or stimulated with anti-CD3 antibody (Clone: OKT3, Biolegend) or treated with 50 ng/ml IL-15 for 4 days. Anti-human CD107a PE was added to and incubated with the culture for 4 h to provoke degranulation before analysis. Three hours before analysis, Brefeldin A was added. Flow cytometry was performed by labeling cells with CD3, CD4, CD28, and CD107a expression was quantified by flow cytometry.

### Co-Culture of CD4^+^CD28^−^ T Cells with Human Renal Glomerular Endothelial Cells

After 24 h of treatment with anti-CD3 antibody and extensive washing, CD4^+^CD28^−^ T cells were co-cultured with human renal glomerular endothelial cells (HRGEC) at a ratio of 1:1 in RPMI 1640 supplemented with 100 mg/ml streptomycin, 100 μg/ml penicillin, and 10% fetal bovine serum (FBS) for 48 h. HREGC were cultured alone or co-cultured with untreated CD4^+^CD28^−^ T cells for 48 h as a control. Cells from the above co-cultures were washed to remove CD4^+^CD28^−^ T cells and stained the remaining adherent HREGC with annexin V-FITC and propidium iodide (PI) and evaluated for apoptosis by flow cytometry conducted with FCAS Canto II (BD Bioscience).

### Multicolour Immunofluorescence Staining

Tissue samples were obtained from patients with LN. These tissue samples were fixed in formalin and paraffin-embedded prior to sectioning. After deparaffinization, the tissue sections were placed in a plastic container filled with Tris–EDTA buffer for antigen retrieval. All sections were incubated with the primary antibodies to GZMB (496B, eBioscience; dilution 1:200) and CD4 (2H4A2, Protein Tech; dilution 1:400) in humidified boxes overnight at 4 °C after blocking with 0.01 M phosphate buffer saline (PBS) containing 3% bovine serum albumin (BSA) for 30 min. Next, the sections were washed and incubated with secondary antibodies (Cy3 conjugated AffiniPure Goat Anti-Rat IgG (GB21302, Servicebio) and FITC conjugated Goat Anti-Mouse IgG (GB22301, Servicebio). The slides were mounted using ProLong Gold antifade reagent with 4′, 6-diamidino-2-phenylindole (DAPI) (G1012, Servicebio) for nuclear staining. Images were acquired with a fluorescence microscope (Leica, Germany).

### Cytokine Assay

Plasma was isolated from fresh EDTA-treated blood samples from SLE patients and healthy controls and stored frozen. Quantitative analysis of IL-5 was performed using enzyme-linked immunosorbent assay (ELISA) (R&D Systems) according to the manufacturer’s instructions.

### Statistics

All quantitative data is presented as mean ± standard deviation (SD). The significance of the differences for independent samples was determined by unpaired two-tailed student’s t-test or two-tailed Mann-Whitney U test. The significance of the differences for paired samples was determined by paired two-tailed student’s t-test or two-tailed Wilcoxon matched-paired signed-rank test. The significance of the differences for samples of more than two groups was determined by two-way ANOVA with Tukey’s multiple comparisons or one-way ANOVA with Tukey’s multiple comparisons. A *P* value less than 0.05 was considered to denote a significant difference.

## RESULTS

### CD4^+^CD28^−^ T Cells with Effector-Memory Cytotoxic Phenotype Abundantly Circulate in SLE Patients and Correlate with SDI

**Fig. 1:**
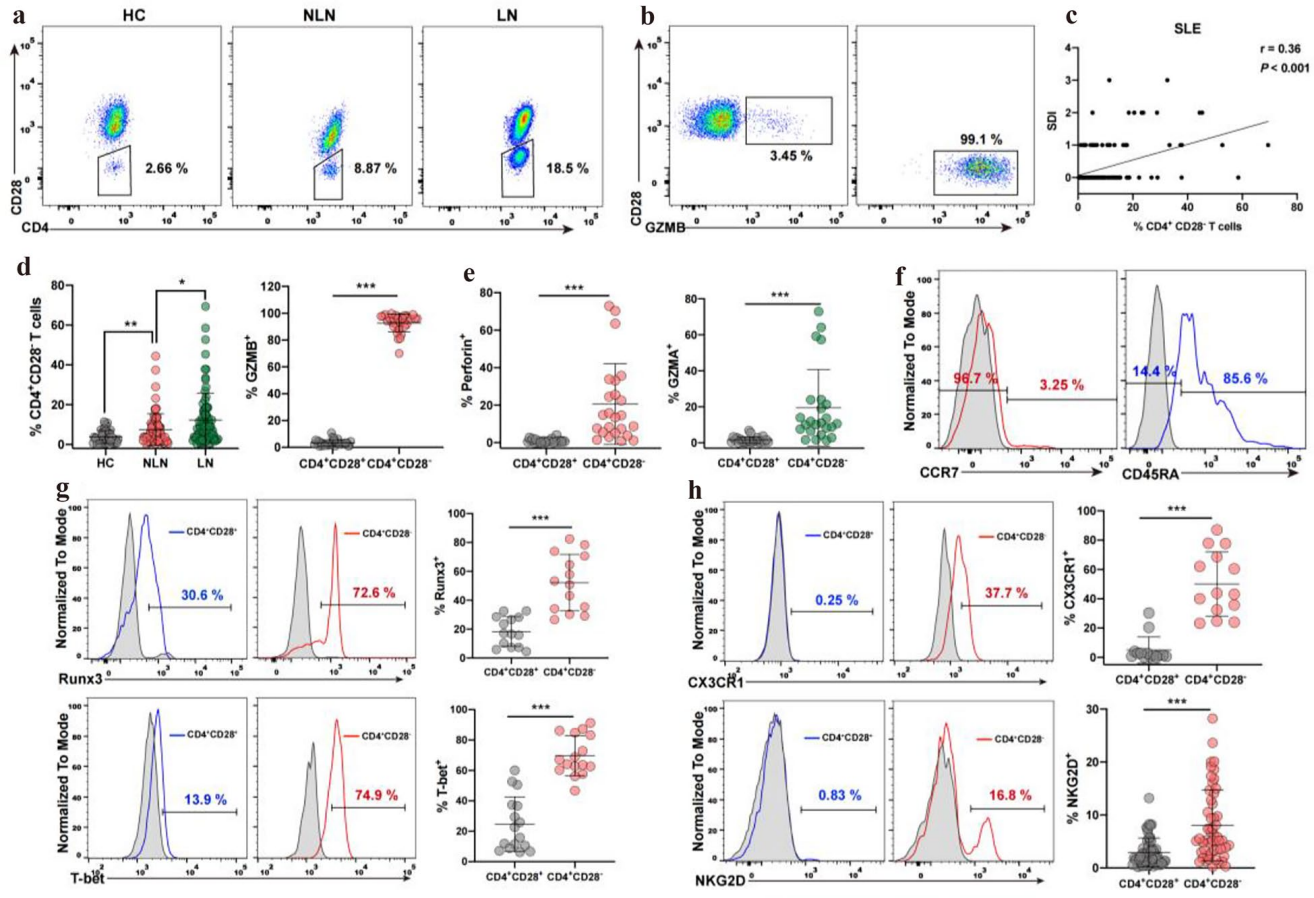
CD4^+^CD28^−^ T cells with effector-memory cytotoxic phenotype are present at high frequency in SLE patients. **A** Flow cytometry determined the frequency of CD4^+^CD28^−^ T cells in PBMCs from HC, NLN (non-lupus nephritis), and LN (Lupus nephritis). **B** Flow cytometry detection of GZMB expression on CD4^+^CD28^−^ T cells and CD4^+^CD28^+^ T cells from SLE. **C** The correlation between the percentage of CD4^+^CD28^−^ T cells and SDI score in SLE is shown. **D** Graphs showing the percentage of CD4^+^CD28^−^ T cells in HC (*n* = 39), NLN-SLE (*n* = 74), LN (*n* = 80), and the percentage of GZMB^+^ cells in CD4^+^CD28^−^ T cells and CD4^+^CD28^+^ T cells from SLE (*n* = 32). **E** Graphs showing the percentage of GZMA + cells (*n* = 24) and perforin + (*n* = 24) in CD4^+^CD28^−^ T cells and CD4^+^CD28^+^ T cells from SLE. **F** Flow cytometry detection of CCR7 and CD45RA expression on CD4^+^CD28^−^ T cells from SLE. **G** Flow cytometry detection of transcription factor Runx3 and T-bet expression on CD4^+^CD28^−^ T cells and CD4^+^CD28^+^ T cells from SLE; graphs showing the percentage of Runx3 + (*n* = 14) and T-bet + (*n* = 16) cells in CD4^+^CD28^−^ T cells and CD4^+^CD28^+^ T cells from SLE. **H** Flow cytometry detection of CX3CR1 and NKG2D expression on CD4^+^CD28^−^ T cells and CD4^+^CD28^+^ T cells from SLE; graphs showing the percentage of CX3CR1 + (*n* = 14) and NKG2D + (*n* = 53) cells in CD4^+^CD28^−^ T cells and CD4^+^CD28^+^ T cells from SLE. Data information: data are presented as mean ± SEM; *P < 0.05, **P < 0.01, ***P < 0.001, two-tailed Mann-Whitney U test (**D**, **E**, **H**) or unpaired two-tailed student’s t-test (**G**).

**Fig. 2:**
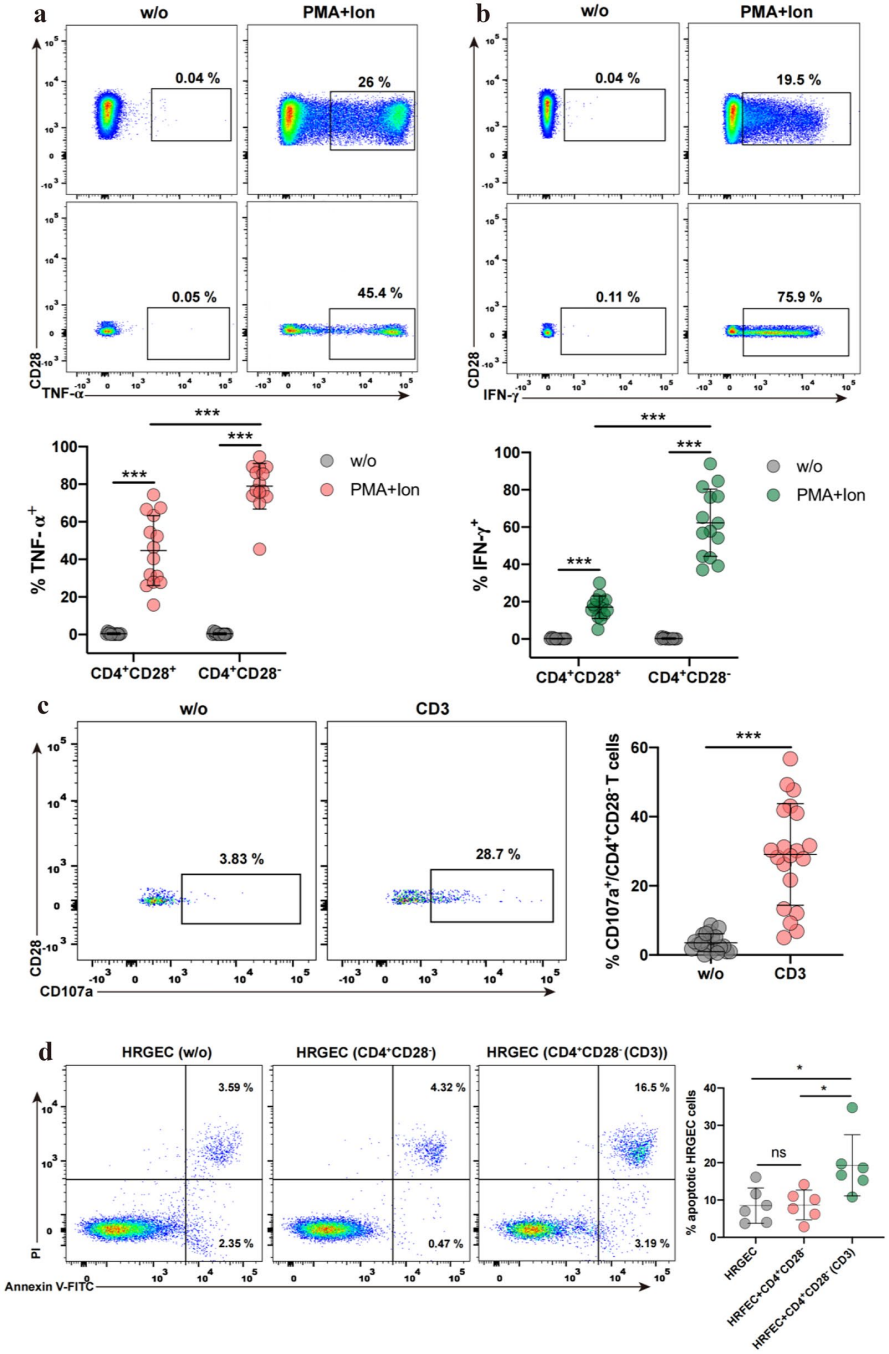
Cytotoxic effect and production of proinflammatory cytokines of CD4^+^CD28^−^ T cells from SLE patients. PBMCs from SLE were cultured alone (w/o) or stimulated with PMA, ion, and brefeldin A for 4 h. Percentages of TNF-α (**A**) and IFN-γ (**B**) in CD4^+^CD28^−^ T cells and CD4^+^CD28^+^ T cells from SLE (*n* = 14) were measured, respectively. **C** PBMCs from SLE were stimulated with an anti-CD3 antibody for 6 h, and degranulation was quantified with CD107a. Percentage of CD107a in CD4^+^CD28^−^ T cells from SLE (*n* = 20) was determined. **D** HRGECs apoptosis induced by anti-CD3 treated CD4^+^CD28^−^ T cells were detected using flow cytometry. Representative histograms show the percentage of apoptotic HRGECs in 48 h co-cultures, without or with CD4^+^CD28^−^ T cells at a ratio of 1:1 in medium with or without anti-CD3 antibody. Graphs show the percentage of apoptotic HRGECs (*n* = 5). Data information: data are presented as mean ± SEM; *P < 0.05, ***P < 0.001. two-way ANOVA with Tukey’s multiple comparisons (**A**, **B**), paired two-tailed student’s t-test (**C**), or one-way ANOVA with Tukey’s multiple comparisons (**D**).

In order to investigate whether CD4^+^ cytotoxic T cells are present in SLE patients, we analyze the frequency of CD4^+^ cytotoxic T cells in blood samples from SLE patients using flow cytometry. As shown in Fig. 1A, D, SLE patients exhibited significantly higher occupancy of CD4^+^CD28^−^ T cells in the circulation than healthy controls. In addition, SLE patients diagnosed with nephritis showed much higher occupancy than those without nephritis. We found that these cells abundantly expressed cytolytic granule Granzyme B (GZMB; 93%), suggesting that these CD4^+^CD28^−^ T cells function as cytotoxic cells (Fig. 1B, D). The percentage of these cytotoxic CD4^+^CD28^−^ T cells positively correlates with SDI (*r* = 0.36, *P* < 0.001), whereas no correlation was found between the percentage of CD4^+^CD28^−^ T cells and SLEDAI (systemic lupus erythematosus Disease Activity Index) (Fig. 1C and Supplementary Fig. 1). We also divided the patients into two different groups, one group without lupus nephritis (NLN) and another group with lupus nephritis (LN) and found that the percentage of CD4^+^CD28^−^ T cells was also positively correlated with SDI after grouping (NLN (*r* = 0.32, *P* = 0.006) and LN (*r* = 0.36, *P* = 0.003). Furthermore, multicolor immunofluorescence studies showed that in patients with lupus nephritis, CD4^+^GZMB^+^ T cells infiltrated into kidney tissues (Supplementary Fig. 2). We also found that a portion of CD4^+^CD28^−^ T cells expressed cytotoxic molecular Granzyme A (GZMA; 20.5%) and Perforin (RPF1; 19.4%) (Fig. 2E). The GZMB/PRF1 and GZMB/GZMA staining profiles showed that the PRF1-expressing and GZMA-expressing CD4^+^CD28^−^ T cells were predominantly GZMB positive, suggesting that cytotoxic granules in CD4^+^CD28^−^ T cells were co-expressed (Supplementary Fig. 3). In addition to the observed increase in granules molecules, the expression levels of transcription factors Runx3 and T-bet were increased in CD4^+^CD28^−^ T cells (Fig. 1G). The cooperation of these two factors is thought to activate the cytotoxic program and contribute to the reprogramming of CD4 Th cells into cytolytic effectors [39]. Furthermore, we found that the chemokine receptor CX3CR1 and NK cell-associated activating receptor NKG2D were also significantly higher in samples from SLE patients compared with those from healthy controls (Fig. 1H). Importantly, these cell subsets showed an effector-memory phenotype evidenced by dual loss of CCR7, CD45RA (TEM), together with re-expressed CD45RA (TEMRA) (Fig. 1F). Together, these results indicated that CD4^+^CD28^−^ T cells from SLE patients are characterized by both an effector-memory state as well as high cytotoxic potential and involved in SLE-related tissue damages.

### CD4^+^CD28^−^ T Cells of SLE Patients Are Functional Cytolytic Cells that Secrete the Cytokines TNF-α and IFN-γ

To investigate the functional attributes of CD4^+^CD28^−^ T cells expanded in SLE patients, we measured their inflammatory cytokine production and cytolytic function. As shown in Fig. 2A, B, after stimulation with PMA/ionomycin, both CD4^+^CD28^−^ T cells and CD4^+^CD28^+^ T cells in SLE patients produced IFN-γ and TNF-α. The frequencies of IFN-γ and TNF-α producing CD4^+^CD28^−^ T cells were much higher than that in CD4^+^CD28^+^ T cells, suggesting that CD4^+^CD28^−^ T cells from SLE patients have the potential signature of proinflammatory cytokine. CD107a has been described as a marker of activation-induced degranulation and is a prerequisite for cytolysis. We next assessed the cytotoxicity of CD4^+^CD28^−^ T cells from SLE patients by quantifying CD107a expressed at the cell surface. We found that activation of CD4^+^CD28^−^ T cells with anti-CD3 antibody increased the expression levels of CD107a (Fig. 2C). In addition, treatment of CD4^+^CD28^−^ T cells with anti-CD3 antibody-induced apoptosis in co-cultured HRGECs, and cd107a expression on the surface of co-cultured CD4^+^CD28^−^ T cells was upregulated (Fig. 2D and Supplementary Fig. 4). Together, these results suggested that cytotoxic CD4^+^CD28^−^ T cells expanded in SLE are functionally active and are capable of producing proinflammatory cytokines.

### IL-15 Triggers Expansion of CD4^+^CD28^−^ T Cells in SLE Patients and Enhances CD4^+^CD28^−^ T Cells into Highly Activated and Cytotoxic Effector T Cells

**Fig. 3:**
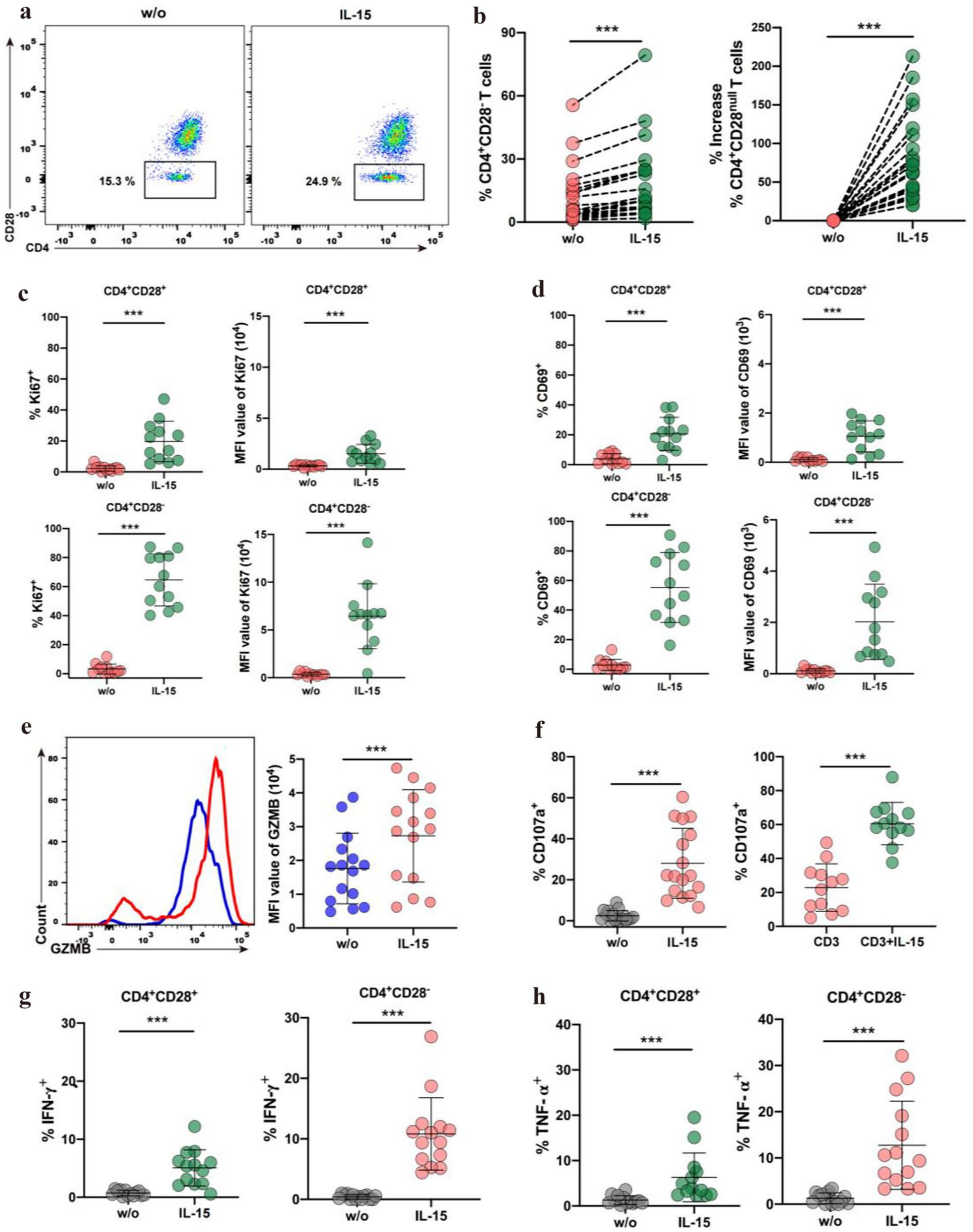
Effects of IL-15 on the expansion and function of CD4^+^CD28^−^ T cells in patients with SLE. PBMCs from SLE were grown in medium alone, with anti-CD3 (1 µg/ml), IL-15 (50 ng/ml), or a combination of both, respectively. **A** Representative dot plots of CD4^+^CD28^−^ T cells (*n* = 23) percentage treated with and without IL-15, respectively. **B** Graphs showing the percentage of CD4^+^CD28^−^ T cells (*n* = 23) and % increase of CD4^+^CD28^−^ T cells. **C** and **D** Graphs showing the percentage of Ki67^+^ (*n* = 12) and CD69^+^ (*n* = 12) cells and the MFI of Ki67 and CD69 in CD4^+^CD28^−^ T cells and CD4^+^CD28^+^ T cells from SLE. **E** Histogram representing the MFI of GZMB on CD4^+^CD28^−^ T cells, the graph shows the MFI of GZMB (*n* = 15) on CD4^+^CD28^−^ T cells cultured without (w/o) and with IL-15. **F** Graphs showing the percentage of CD107a^+^ cells (*n* = 17) on CD4^+^CD28^−^ T cells cultured without (w/o) and with IL-15 and the percentage of CD107a^+^ cells (*n* = 12) cultured with anti-CD3 or anti-CD3 + IL-15. **E** Graphs show the percentage of IFN-γ^+^ cells (*n* = 14) on CD4^+^CD28^+^ and CD4^+^CD28^−^ T cells cultured without (w/o) and with IL-15. **H** Graphs showing the percentage of TNF-α^+^ cells (*n* = 14) on CD4^+^CD28^−^ and CD4^+^CD28^+^ T cells cultured without (w/o) and with IL-15. Data information: data are presented as mean ± SEM; ***P < 0.001, paired two-tailed student’s t-test (**B**, **C**, **D**, **E**, and **F**) or (**G**, **H**).

While we successfully validated and characterized CD4^+^ cytotoxic T cells in our study, evidence for their existence remains circumferential, with little direct evidence of how these cells expand or function in the pathogenesis of SLE. Previous studies have shown that the cytokine IL-15 is overproduced in some inflammatory and autoimmune diseases. Chronic exposure to IL-15 may promote the expansion of CD4^+^CD28^−^ T cells *in vivo* [40–42]. Here, we investigated whether IL-15 is involved in the expansion and regulates the function of cytotoxic CD4^+^CD28^−^ T cells in SLE. Our results showed that IL-15 was increased in the plasma of SLE patients (Supplementary Fig. 5). Furthermore, incubation in IL-15 significantly increased the frequency of CD4^+^CD28^−^ T cells in SLE patients (Fig. 3A, B). Next, we investigated the effects of IL-15 on the proliferation of CD4^+^CD28^−^ T cells in SLE patients by determining the expression of Ki67, a well-known proliferation marker for cell proliferation evaluation. The results showed that Ki67-expressed CD4^+^CD28^−^ T cells and the expression level (based on mean fluorescence intensity (MFI)) of Ki67 on CD4^+^CD28^−^ T cells were both significantly increased after stimulation with IL-15. In contrast, minor changes in Ki67-expressed cells and Ki67 expression level were observed in the CD4^+^CD28^+^ T cells followed by IL-15 treatment (Fig. 3C and Supplementary Fig. 6A). To identify the active status of CD4^+^CD28^−^ T cells, we measured the expression level of the activation marker CD69. As shown in Fig. 3D and Supplementary Fig. 6B, treatment with IL-15 significantly increased the proportion of CD69-expressing CD4^+^CD28^−^ T cells and the CD69 expression levels (MFI) on CD4^+^CD28^−^ T cells. We observed only minor differences in the CD69 expression of the CD4^+^CD28^+^ T cells counterparts. In addition, we assessed the effects of IL-15 treatment on the expression of granzyme B in CD4^+^CD28^−^ T cells from SLE patients. As shown in Fig. 3E and Supplementary Fig. 6C, IL-15 did increase the expression levels (MFI) of granzyme B in CD4^+^CD28^−^ T cells, although it did not change the percentage of CD4^+^CD28^−^ T cells expressing granzyme B. Next, we investigated whether the release of cytotoxic molecules was affected by IL-15 using a degranulation assay based on the CD107a expression. Our results showed that IL-15 significantly upregulated the degranulation of CD4^+^CD28^−^ T cells. Moreover, IL-15 significantly upregulated the anti-CD3-induced degranulation of CD4^+^CD28^−^ T cells (Fig. 3F and Supplementary Fig. 6D). We also found that IL-15 induces the production of disease-related proinflammatory cytokines in CD4^+^CD28^−^ T cells. The percentage of cells that produce TNF-α and IFN-γ significantly increased in both CD4^+^CD28^−^ and CD4^+^CD28^+^ T cells treated with IL-15 (Fig. 3G, H and Supplementary Fig. 6E), and the increase was higher in CD4^+^CD28^−^ T cells compared to CD4^+^CD28^+^ T cells. Together, these results suggest that following IL-15 treatment, CD4^+^CD28^−^ T cells develop a higher proliferative capacity and higher activity status than their CD4^+^CD28^+^ T cells counterparts.

### IL-15 Activates JAK3/STAT5 Signaling Pathway in CD4^+^CD28^−^ T Cells from SLE Patients

**Fig. 4:**
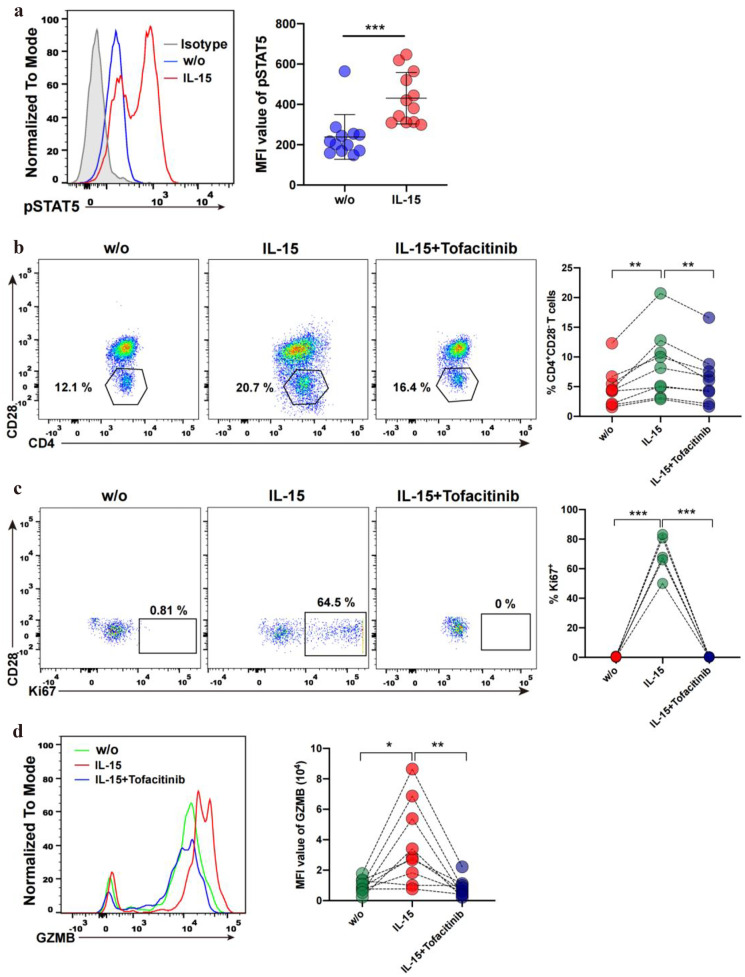
IL-15 activates JAK3/STAT5 pathway in CD4^+^CD28^−^ T cells from SLE patients. **A** Phosphorylation levels of STAT5 were determined in CD4^+^CD28^−^ T cells cultured without (w/o) or with IL-15. Histogram and graph showing the phosphorylation levels of STAT5 (*n* = 12). Dark-shaded histograms indicate negative isotypic control. **B** Representative dot plots of CD4^+^CD28^−^ T cells percentage, graphs showing the percentage of CD4^+^CD28^−^ T cells (*n* = 9) untreated (w/o), treated with IL-15 or IL-15 plus Tofacitinib. **C** Representative dot plots of Ki67 expressing on CD4^+^CD28^−^ T cells, graphs showing the percentage of Ki67^+^ (*n* = 5) in CD4^+^CD28^−^ T cells untreated (w/o), treated with IL-15 or IL-15 plus tofacitinib. **D** Histogram representing the MFI of GZMB on CD4^+^CD28^−^ T cells, the graph shows the MFI of GZMB (*n* = 9) on CD4^+^CD28^−^ T cells treated with nothing (w/o), IL-15 and IL-15 plus tofacitinib. Data information: data are presented as mean ± SEM; *P < 0.05, **P < 0.01,***P < 0.001, two-tailed Wilcoxon matched-paired signed-rank test (**A**) or paired two-tailed student’s t-test (**B**, **C**, **D**).

Previous studies showed that IL-15 receptor signaling is transmitted through the JAK/STAT and RAS/Raf/ERK signaling pathway [43–45]. In order to investigate the mechanism underlying IL-15 regulated function of CD4^+^CD28^−^ T cells, we analyzed the activity of components involved in the IL-15 receptor signaling pathways. As shown in Fig. 4A, engagement of IL-15R with IL-15 induced an increase of STAT5 phosphorylation in CD4^+^CD28^−^ T cells. In contrast, phosphorylated ERK levels changed little in the same condition (Supplementary Fig. 6). Next, we block IL-15 signaling with the JAK3 selective inhibitor tofacitinib. As shown in Fig. 4B, the expansion and proliferation of CD4^+^CD28^−^ T cells were inhibited after adding tofacitinib. In addition, our results show that blocking IL-15 signaling with tofacitinib also decreased the percentage of Ki67-expressed CD4^+^CD28^−^ T cells and the expression level (MFI) of GZMB in CD4^+^CD28^−^ T cells (Fig. 4C, D). Together, these findings suggested that IL-15 regulates the expansion, proliferation, and effector function of CD4^+^CD28^−^ T cells from SLE patients through activation of the JAK3/STAT5 and NKG2D signaling pathway.

### IL-15 Upregulates NKG2D Expression and Activates the NKG2D Pathway in CD4^+^CD28^−^ T Cells from SLE Patients

**Fig. 5:**
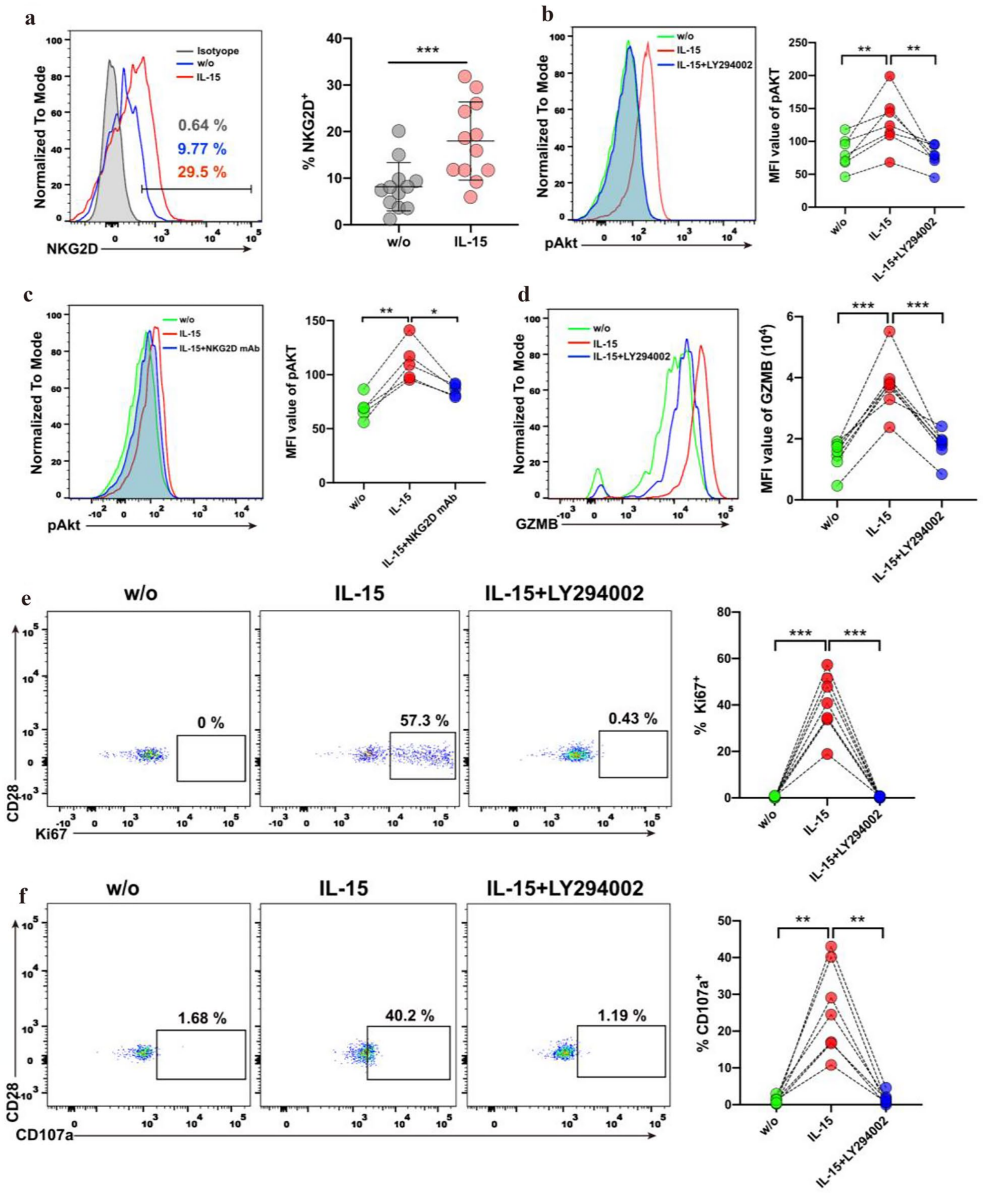
IL-15 upregulates NKG2D expression and activates the NKG2D pathway in CD4^+^CD28^−^ T cells from SLE patients. **A** Representative histogram for NKG2D expression on CD4^+^CD28^−^ T cells, the graph shows the percentage of NKG2D^+^ cells (*n* = 12) on CD4^+^CD28^−^ T cells cultured without (w/o) and with IL-15. Dark-shaded histograms indicate negative isotypic control. **B** Phosphorylation levels of Akt were determined in CD4^+^CD28^−^ T cells cultured in medium alone (w/o), with IL-15 or IL-15 plus LY294002. Histogram and graph showing the phosphorylation levels of Akt (*n* = 6). **C** Phosphorylation levels of Akt were determined in CD4^+^CD28^−^ T cells cultured in medium alone (w/o), with IL-15 or IL-15 plus NKG2D neutralizing antibody. Histogram and graph showing the phosphorylation levels of Akt (*n* = 6). **D** Histogram representing the MFI of GZMB on CD4^+^CD28^−^ T cells, the graph shows the MFI of GZMB (*n* = 7) on CD4^+^CD28^−^ T cells treated with nothing (w/o), IL-15 and IL-15 plus LY294002. **E** Representative dot plots of Ki67 expressing on CD4^+^CD28^−^ T cells, graphs showing the percentage of Ki67^+^ (*n* = 7) in CD4^+^CD28^−^ T cells untreated (w/o), treated with IL-15 or IL-15 plus LY294002. **F** Representative dot plots of CD107a expressing on CD4^+^CD28^−^ T cells, graphs showing the percentage of CD107a^+^ (*n* = 7) in CD4^+^CD28^−^ T cells untreated (w/o), treated with IL-15 or IL-15 plus LY294002. Data information: data are presented as mean ± SEM; **P < 0.01,***P < 0.001, paired two-tailed student’s t-test (**A**, **B**, **C**, **D**, **E**, and **F**).

We have shown increased surface NKG2D on CD4^+^CD28^−^ T cells compared to CD4^+^CD28^+^ T cells in Fig. 1H. And previous studies showed that IL-15 increases the abundance of NKG2D and DAP10 in the NK cell surface membrane *in vitro*, which primes the cytotoxic responses of NK cells [46–48]. Here, we investigate whether IL-15 cooperatively initiates the NKG2D pathway to regulate CD4^+^CD28^−^ T cell cytotoxicity. As shown in Fig. 5A, we found that the expression of surface NKG2D on CD4^+^CD28^−^ T cells was upregulated after exposure to IL-15. To evaluate whether IL-15 induces the activation of the NKG2D-mediated signaling pathway, we assessed the phosphorylation events downstream of the NKG2D pathway. As shown in Fig. 5B, we found that AKT phosphorylation levels were significantly increased in CD4^+^CD28^−^ T cells following the addition of IL-15 and pretreatment of CD4^+^CD28^−^ T cells with the PI3K inhibitor LY294002 inhibited AKT phosphorylation. However, IL-15R engagement also mediates PI3K/AKT pathway activation. Thus, to further investigate whether the phosphorylation of AKT is completely dependent on NKG2D/DAP10 signaling pathway, we blocked the NKG2D with neutralizing antibody and analyzed the phosphorylation level of AKT in CD4^+^CD28^−^ T cells treated with IL-15. We observed that treatment of CD4^+^CD28^−^ T cells with IL-15 plus NKG2D neutralizing antibody partially inhibited the phosphorylation of AKT, indicating that IL-15 combined with NKG2D activate the PI3K/AKT pathway (Fig. 5C). Furthermore, our results show that pretreatment of CD4^+^CD28^−^ T cells with the PI3K inhibitor LY294002 significantly decreased the percentage of Ki67-expressed CD4^+^CD28^−^ T cells and CD107a-expressed CD4^+^CD28^−^ T cells and the expression level (MFI) of GZMB in CD4^+^CD28^−^ T cells (Fig. 5D–F). Together, these results suggested that IL-15 stimulation induces the activation of NKG2D-mediated signaling pathway in CD4^+^CD28^−^ T cells and the consequent phosphorylation of AKT.

## DISCUSSION

In this study, we systematically analyzed the phenotypic features and cytolytic functions of CD4^+^CD28^−^ T cells in the peripheral blood of SLE patients and identified the effects of IL-15 on the expansion, proliferation, and cytotoxic function of CD4^+^CD28^−^ T cells and its mechanism. Our study demonstrated that IL-15 combined with the NKG2D pathway triggers the expansion and cytotoxic function of CD4^+^CD28^−^ T cells, predominantly via the JAK3/STAT5 and PI3K/AKT signaling pathway.

A previous study reported that cytotoxic CD4^+^NKG2D^+^ T cells expand in SLE patients and showed that this population secretes granzyme and perforin, thus inducing apoptosis of the Treg cells [49]. Here, we demonstrated that the number of cytotoxic CD4^+^CD28^−^ T cells increases in the peripheral blood of SLE patients, especially in patients with lupus nephritis. The percentage of CD4^+^CD28^−^ T cells in SLE patients significantly correlated with SDI score, indicating that CD4^+^CD28^−^ T cells also exert pathogenic function and are involved in SLE-disease tissue damages. In addition, we found that these cells infiltrate the renal tissue of patients with lupus nephritis, displaying direct cytotoxic activity against HRGECs i after anti-CD3 antibody treatment. The result has been confirmed in the recently published article, and the study revealed that excessive IL-15 promotes cytotoxic CD4^+^CD28 − T cell-mediated injury in human glomerular endothelial cells (GEnCs) in lupus nephritis [50]. Furthermore, our results showed that CD4^+^CD28^−^ T cells from SLE patients functioned more as a proinflammatory phenotype than their CD28^+^ counterparts, because the former cell produces higher levels of IFN-γ and TNF-α. Overall, these results suggest that CD4^+^CD28^−^ T cells are characterized by cytotoxic potential and are involved in renal tissue injury in SLE patients.

IL-15 is a crucial manipulator of T-cell function and has been found to promote T-cell survival, proliferation, and influence effector functions. However, whether IL-15 plays a role in SLE is less established. Previous studies have shown that serum IL-15 levels are elevated in SLE patients. This increase has previously been shown to be especially high in patients suffering from lupus nephritis [51, 52]. In agreement with these earlier studies, we found that IL-15 levels in SLE patients’ plasma were higher than those in healthy controls. Furthermore, we found that cytokine IL-15 plays a critical role in regulating the activation and function of CD4^+^CD28^−^ T cells from SLE patients. We dissected the mechanistic basis of IL-15 effects on CD4^+^CD28^−^ T cells from SLE patients and demonstrated that the activation of IL-15/IL-15R/JAK3/STAT5 pathway with the higher levels of phosphorylated STAT5 in CD4^+^CD28^−^ T cells. Tofacitinib, a selective JAK3 inhibitor that blocks IL-15 signaling, significantly prevents the expansion, proliferation, and cytotoxic function of CD4^+^CD28^−^ T cells. Our results suggested that the IL-15/IL-15R/JAK3/STAT5 pathway is a crucial molecular pathway for IL-15 to regulate the function of CD4^+^CD28^−^ T cells in SLE patients. In addition, our study also found that the expression of NKG2D is upregulated in CD4^+^CD28^−^ T cells as a result of IL-15 treatment. An earlier report claimed that IL-15 upregulates the expression and function of NKG2D on CD28^−^negative effector CD8 CTL [46]. Another study indicated that the IL-15 receptor signaling pathway couples with the NKG2D receptor signaling pathway, thus priming NK cells’ survival, proliferation, and cytotoxic responses through JAK3-mediated phosphorylation of DAP10 [53]. The associated adaptor DAP10 mediates NKG2D signaling through a YXXM motif that binds and activates the vav1 and p85 subunits of PI3K [54]. On the basis of these results, we further investigated whether the activated IL-15 receptor cooperates with the NKG2D signaling pathway to regulate the function of CD4^+^CD28^−^ T cells. Our results showed that the activation of the PI3K/AKT pathway in CD4^+^CD28^−^ T cells following IL-15 addition, and pretreatment of CD4^+^CD28^−^ T cells with the PI3K inhibitor LY294002 inhibited AKT phosphorylation. However, previous studies revealed that the P3K/AKT/mTOR pathway is also critical for the IL-15-mediated activation of NK cells [55, 56]. Thus, we attempted to show the association between the activation of PI3K/AKT pathway, IL-15, and NKG2D by blocking CD4^+^CD28^−^ T cells with NKG2D neutralizing antibody before treating them with IL-15. We found that the crosstalk between IL-15R and NKG2D contributes to the phosphorylation of downstream AKT signaling. Meanwhile, the PI3K inhibitor LY294002 significantly prevents the proliferation and cytotoxic function of CD4^+^CD28^−^ T cells.

In conclusion, our study demonstrated that CD4^+^CD28^−^ T cells play a critical role in the progression of inflammatory disorders and tissue damage. Importantly, our results suggested that IL-15 contributes to the pathogenesis of SLE by inducing the activation of CD4^+^CD28^−^ T cells via the JAK3/STAT5 signaling pathway. Our study also showed that coordinated priming of the NKG2D pathway by IL-15 induced CD4^+^CD28^−^ T cell cytotoxicity. The PI3K/AKT pathway mediated by both IL-15 and NKG2D drives the development, proliferation, and effector functions of CD4^+^CD28^−^ T cells. In addition, further investigation should be done to fully elucidate the specific transcriptional mechanisms of IL-15 signaling that regulate the expression of genetic programs of CD4^+^CD28^−^ T cells.

### Supplementary Information

Below is the link to the electronic supplementary material.

Supplementary file1 (DOCX 3479 KB)

## Data Availability

All data in this study are included in this published article.
